# Knowledge and awareness about the harmful effects of sun exposure on the skin and sun safety methods used in the adult Kuwaiti population: A questionnaire-based study

**DOI:** 10.1016/j.jdin.2025.05.021

**Published:** 2025-08-22

**Authors:** Rawan Almutairi, Atlal Allafi

**Affiliations:** Dermatology Department, As’ad Alhamad Dermatological Center, Kuwait

**Keywords:** awareness, knowledge, non-melanoma skin cancer, skin cancer, sunscreen, ultraviolet

## Abstract

**Background:**

Sun exposure can have both positive and negative effects on human health.

**Objective:**

This study aimed to evaluate the awareness level about sun harmful effects on the skin of adult Kuwaitis and to study their attitudes and practices of using sun-protective methods.

**Methods:**

Observational multicentric questionnaire study was conducted on patients who visited primary health care centers in Kuwait. Data were collected from 2023 to 2024. The sample included only Kuwaiti adults aged 18 and older.

**Results:**

723 individuals participated in this study. The mean age was 36 years. Two-thirds of the participants were females. Almost two-thirds of respondents were both mindful of the association between sun exposure and skin aging and pigmentation, while minority reported no side effects. Thirty-nine percent of respondents were aware of the association between skin cancer and solar exposure. Females and lighter skin type demonstrated a higher level of using sunscreens. Only 27% of the participants were using sunscreens regularly and almost half of those who did not use it stated that it is not important to apply sunscreen.

**Conclusion:**

Kuwaiti adults possessed a satisfactory awareness level regarding sun adverse effects. The utilization of sunscreen was inadequate, regardless of their awareness and education.

**Capsule Summary**

• The Kuwaiti population's awareness regarding the implementation of solar protection measures is not well-documented.
• To assess the degree of awareness among adult Kuwaitis regarding the adverse effects of solar exposure on their skin, as well as to investigate their attitudes and behaviors regarding sun protection.

## Introduction

Skin damage and many skin diseases are unavoidable consequences of excessive unprotected sun exposure.^1^ Short-term ultraviolet (UV) exposure leads to acute damage, including burning and tanning, while long-term UV exposure may result in chronic skin diseases, such as pigmentary changes in solar lentigines, ephelides, and melasma. Furthermore, long-term UV exposure can cause skin cancer and aging, including telangiectasias and elastosis.^2^

All skin layers experience changes related to UV exposure, with the majority occurring in the epidermis and dermis. In the epidermis, there is a loss of Langerhans cells and the presence of dyskeratotic cells (ie, sunburn cells). While the dermis is noted to have enlarged endothelial cells and edema caused by mast cell degranulation. The degranulation started within 30 minutes of exposure to UV radiation. These skin changes were reversed 24 h after exposure to UV radiation.^3^ Furthermore, the survived cells with less severe damage may have deoxyribonucleic acid mutations that could ultimately contribute to the onset of skin cancers.^4^

Skin cancer is the most common type of cancer worldwide, and is divided into melanoma and non-melanoma skin cancer (NMSC). The annual incidence of NMSC is consistently increasing, and it is the most prevalent cancer in the United States. Between 1992 and 2006, the number of annual NMSC cases in the Medicare population in the United States increased by an average of 4.2%.^5^ The incidence of both melanoma and NMSC in later life has been correlated with sun exposure in early life. Hence, UV radiation is the most important modifiable risk factor for the development of this tumor.^6^^,^^7^

Sunscreens block the transfer of UV radiation into the epidermis by reflecting, absorbing, or scattering it. Its application to human skin reduces the acute effects of UV radiation on the appearance of sunburn cells, migration of Langerhans cells out of the epidermis, and inflammatory response. Consequently, sunscreens have been recommended as a method of defense against the harmful effects of radiation, with the level of protection increasing as the sun protection factor increases.^8^

As the Kuwaiti population's awareness regarding the implementation of solar protection measures is not well-documented, this study was conducted to assess the degree of awareness among adult Kuwaitis regarding the adverse effects of solar exposure on their skin, as well as to investigate their attitudes and behaviors regarding sun protection.

## Materials and methods

### Study design and setting

An observational multicenter cross-sectional questionnaire-based study was conducted on both males and females who attended primary health care centers in the six governmental areas in Kuwait (Al Asimah, Al Jahra, Hawalli, Farwaniyah, Mubarak Al-Kabeer, and Al-Ahmadi). From each governorate, one major city was selected. Data were collected from February 2023 to February 2024.

### Data collection

Self-completed surveys were distributed to primary health care outpatients who were visiting the clinics for various purposes. The sample representing the general public was randomly selected to participate in the survey. The authors formulated the questions following a literature review of solar awareness surveys conducted in other nations.^9^, ^10^, ^11^, ^12^, ^13^, ^14^ The final self-administered questionnaire consisted of 22 questions and took approximately 5 minutes to complete. The questionnaire utilized for data collection was evaluated on a pilot group of 15 individuals in order to determine the approximate completion time. The concluding analysis did not include these questionnaires. Cognitive testing of the questionnaire was performed to assess its validity and comprehensibility.

### Study sample

In 2023, the population of Kuwaiti above 18 years of age (based on the Public Authority of Civil Information database) was 3,230,650 comprising 1,033,808 Kuwaitis and 2,196,842 non-Kuwaitis living in the six governorates. To estimate sample size of 35% of the Kuwaiti population (1,033,808) with a sample error of ±3% and 95% confidence level, at least 971 individuals needed to be interviewed. The sample included Kuwaiti adults aged 18 and older, and excluded those non-Kuwaiti and those younger than 18.

### Data analysis

The data were entered into Microsoft Excel and then exported to Statistical Package for the Social Sciences version 25 for descriptive analysis. Quantitative data were analyzed using the mean and standard deviation, while qualitative data were analyzed using descriptive statistics in the form of frequency and percentage. The *X*^2^ test was used as a non-parametric test of significance for comparing the distributions of two qualitative variables. Statistical significance was defined as a *P* < .05. The independent predictive effects of demographic variables on the knowledge of solar damage and the use of sunscreens were investigated using logistic regression.

### Ethical approval

Ethical approval for the study was obtained from standing committee for coordination of medical research, Ministry of health, Kuwait (2239/2023).

## Results

### Sociodemographic variables

In total, 723 individuals agreed to participate in the study, with a response rate of 74.5%. The background characteristics of the study population are outlined in Table I. The age distribution of the participants was varied. The mean age of the participants was 36 years with standard deviation 9.1 years (range: 18-68, median: 35 years). Approximately two-thirds of the participants were female (60%). Approximately two-thirds of participants were married (62%). Regarding education, 89% and 11% of participants were postgraduates and graduates, respectively. Half of the study participants had a medium socioeconomic status, followed by high (36.9%) and low (13%). Skin types 3 and 4 were the most common skin types reported in this study, representing 81% of individual skin types. The majority of participants had indoor occupations (91.3%), and a minority had a family history of skin cancer (2.2%). When asked about the history of sunburn, 287 individuals (39.7%) had a positive history.

**Table I:** Personal and demographic characteristics of the study population

	*n* = 723	%
Age (mean ± SD, y)	36 ± 9.1	-
Gender		
Female	434	60.0
Male	289	40.0
Marital status		
Married	454	62.8
Single	269	37.2
Education		
Post graduate	78	10.8
Graduate	645	89.2
Socioeconomic status		
High	267	36.9
Medium	362	50.1
Low	94	13.0
Fitzpatrick skin type		
Type I	0	0.0
Type II	94	13.0
Type III	188	26.0
Type IV	401	55.5
Type V	20	2.8
Type VI	20	2.8
Occupation		
Indoor	660	91.3
Outdoor	63	8.7
Family history of skin cancer	16	2.2
History of sunburn		
No	436	60.3
Yes, once	279	38.6
Yes, more than once	8	1.1

### Sun protection behavior

Table II shows the respondents’ sun-protective behavior. Approximately 75% of the respondents did not regularly use sunscreen products during their outdoor activities, and a minority (16%) regularly used hats. The majority of the respondents stayed in shade (89.3%) and wore sunglasses (72.6%). Those who wore long-sleeved clothes regularly represented three-quarters of the study population.

**Table II:** Sun-protective behaviors among 723 respondents

	Regularly *n* (%)	Sometimes *n* (%)	Never *n* (%)
Use sunscreen products	195 (27)	262 (36.2)	266 (36.8)
Hats	120 (16.6)	173 (23.9)	430 (59.5)
Long-sleeved clothes	541 (74.8)	127 (17.6)	55 (7.6)
Stay in shade	195 (27)	451 (62.4)	77 (10.7)
Sunglass	249 (34.4)	276 (38.2)	198 (27.4)

### Sun exposure knowledge

Education, skin type, socioeconomic status, marital status, and location of workplace had associations with sun exposure knowledge. Table III illustrates respondents' knowledge of the consequences of unprotected sun exposure on their skin (participants can select more than one option). Approximately two-thirds of the respondents were aware of the correlation between UV exposure and skin aging (60%) and pigmentation (68.9%). The majority of participants were aware of sun-induced skin tanning (85.9%), while a minority reported no side effects of the sun on the skin (2%). Most of the respondents (76.3%) understood the link between sun exposure and skin burns. A relationship between skin cancer and solar exposure was observed in 39% of the participants. The logistic regression of the knowledge of sun harmful effects is presented in Table IV.

**Table III:** Participants’ knowledge of skin damage induced by sun exposure

	*n* = 723 (%)
Skin darkening	621 (85.9)
Skin burn	552 (76.3)
Skin pigmentation	498 (68.9)
Skin aging	434 (60.0)
Skin cancer	280 (38.7)
Has no side effects	15 (2.1)

**Table IV:** Multiple logistic regressions of the knowledge of sun harmful effects and sociodemographic characteristics

	Odds ratio (95% CI)	*P* value
Sunburn		
Postgraduates	9.26 (4.19-20.43)	<.001
Skin cancer		
Skin type 4	2.73 (1.8-4.14)	<.001
Skin darkening		
High socioeconomic status	5.99 (2.76-13.02)	<.001
Married	3.49 (1.79-6.8)	<.001
Skin aging		
Indoor Workers	6.68 (2.83-15.77)	<.001
Postgraduates	5.1 (2.3-11.27)	<.001

### Behavior of sunscreen use

Gender, skin type, socioeconomic status, and history of sunburn had associations with the use of sunscreen. Table V shows the behavior of using sunscreen among regular and sometimes sunscreen users (*n* = 457). The majority of them apply sunscreen with a sun protection factor equal to or greater than 30. Morning time was the preferred application time (84.2%). Regarding the sites of application of sunscreen products, both face and hands represent the most common sites of application (94.7% and 51.9%, respectively). Most sunscreen users agreed to use sunscreen during both the winter and summer (70%).

**Table V:** Behaviors of using sunscreen products among regular and sometimes sunscreen users (*n* = 457)

	*n* = 457 (%)
Sun protection factor used	
≥30	322 (70.5)
˂30	135 (29.5)
Time of application	
Morning only	385 (84.2)
Afternoon only	13 (2.8)
Both morning and afternoon	59 (12.9)
Site of application	
Face	433 (94.7)
Hands	237 (51.9)
Neck	68 (14.9)
Season of application	
Summer only	102 (22.3)
Winter only	35 (7.7)
Both summer and winter	320 (70)

**Fig 1:**
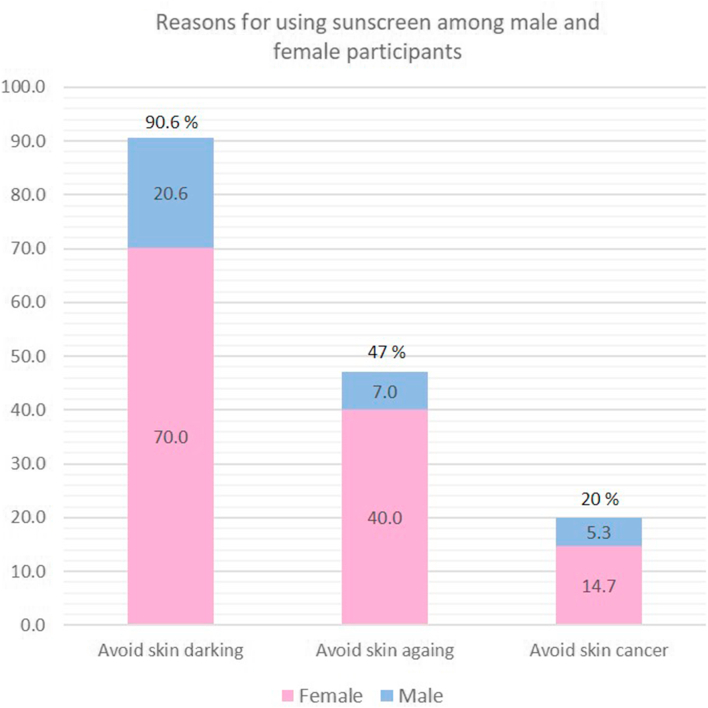
Reasons for using sunscreen among male and female participants. The bar chart shows the proportion of participants reporting sunscreen use to avoid skin darkening, skin aging, and skin cancer. Female participants reported higher use across all three reasons compared to males.

**Fig 2:**
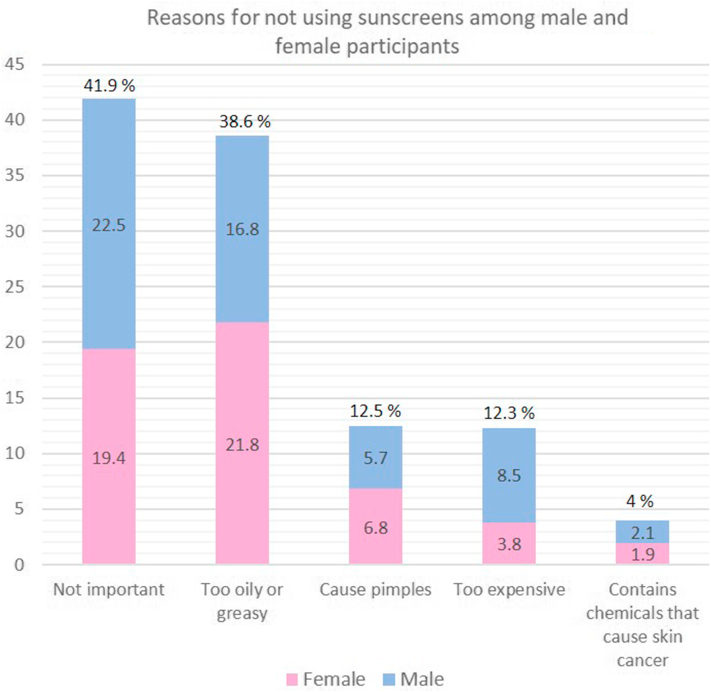
Reasons for not using sunscreen among male and female participants. Commonly reported barriers were lack of importance, oily or greasy texture, pimples, high cost, and concerns about harmful chemicals.

Females were significantly more likely to use sunscreen products than males (OR = 19.81, 95% CI [9.2-42.64], *P* < .001). Additionally, participants with lighter skin type (type 2) demonstrated a higher level of use of sunscreen products (OR = 34.8, 95% CI [9.08-133.77], *P* < .001). The logistic regression for sunscreen use is presented in Table VI. These associations were not influenced by any other social or demographic factors. The reasons for using or not using sunscreens among the study participants are shown in Figs 1 and 2.

**Table VI:** Multiple logistic regressions on sunscreen use and sociodemographic characteristics

	Odds ratio (95% CI)	*P* value
Females	6.23 (3.88-10.02)	<.001
Skin type 2	37.71 (10.03-141.78)	<.001
High socioeconomic status	16.69 (4.95-56.26)	<.001
History of sunburn	3.19 (1.97-5.16)	<.001

## Discussion

To our knowledge and after an extensive literature review, this is the first multicentric population-based study in Kuwait to evaluate the prevalence of sunscreen use, and other sun-protective methods, and to study the factors associated with using sunscreen among Kuwaiti adults, as well as evaluate their knowledge of the harmful effects of sun exposure, and compare our results with regional and international populations.

Kuwait experiences extreme temperatures, particularly during the summer months. Daytime highs can reach 48 °C in July and August. The UV index in Kuwait is also notably high, often exceeding levels considered safe for prolonged outdoor activities. For instance, during the summer months, the UV index can reach values as high as 10, categorized as “very high” or even “extreme” on certain days.^15^

In Kuwait, NMSC, including basal cell carcinoma and squamous cell carcinoma, are primarily diagnosed in men with a male-to-female ratio of 2.3:1.^1^ Although a high number of skin cancers were detected in men, our study showed that men were less likely than women to use sunscreens. The two most reported reasons for not using sunscreen among male participants were that the skin was too oily and unimportant. Women were six times more likely to apply sunscreen than males, suggesting that they are more cognisant of the health risks associated with UV radiation exposure. Our results were in line with those of Australia,^9^ the world’s highest country for skin cancer, Saudi Arabia,^10^ Turkey,^11^ and Malta.^12^ The literature has reported a number of factors that are positively correlated with the use of sunscreen. The most significant factor is female gender, followed by higher income, greater education, and a lighter skin color.^13^ We found that multiple barrier methods of sun protection such as hats and long-sleeved shirts are not frequently utilized. This is despite knowledge that sunscreen alone is unable to protect an individual entirely from UV radiation exposure.^16^ In line with the results of Basch et al,^17^ respondents with darker skin were less inclined to utilize sunscreens than those with lighter skin (skin type II). One explanation is that those with darker skin phenotypes feel safe because of their darker natural skin type. However, this may lead to an overestimation of the skin’s self-protection against solar UV radiation, which in turn can increase the risk of sunburns and the development of melanoma and NMSC.

The majority of the volunteers concurred that sun exposure resulted in skin darkening, burns, pigmentation, and aging. However, only 38% of our volunteers were aware that sun exposure could cause skin cancer, in contrast to 98% of the volunteers in another study conducted in Spain.^14^ In Saudi Arabia, there was also a poor level of knowledge of the risk of developing skin cancer as a result of sun exposure (16.7%).^18^ Despite the good understanding and familiarity among our study respondents that exposure to the sun predisposes individuals to a variety of skin disorders, the rate of sunscreen interest was relatively low.

A higher socioeconomic status is frequently associated with enhanced access to information and resources, which can contribute to an increased understanding of skin cancer and solar exposure. Individuals in higher socioeconomic level may have greater access to healthcare and educational materials about the risks of sun exposure, leading to an improved awareness of necessary protective measures.^19^

There are numerous strategies that can enhance the education on skin cancer for individuals who are less educated or have misconceptions. For example, it is imperative to emphasize that sunscreen is essential regardless of whether it is cloudy or whether one is working indoors. Additionally, ensure that educational resources are available in locations where individuals gather, such as community centers, clinics, or schools, to ensure that information is disseminated to a broader audience.

In Kuwait, cultural norms and traditions significantly influence clothing choices, often resulting in greater coverage, particularly for women. This degree of coverage may result in a misunderstanding regarding the necessity of sunblocks, as many individuals may believe that clothing offers adequate protection from damaging ultraviolet radiation. However, it is important to note that, even when covered, the skin can still be exposed to UV radiation, particularly in areas such as the face and hands. In addition, certain fabrics may not effectively block UV radiation, particularly if they are lightweight. Skin protection awareness could be improved by providing education on the significance of sunscreens, irrespective of the level of clothing coverage. Thus, the cultural context plays a crucial role in shaping attitudes toward sunscreen use and could benefit from targeted public health messaging that respects these traditions while promoting skin health.

In general, sunscreen was utilized by only 27% of the participants, and nearly half of those who did not apply sunscreen believed that it was unnecessary. Therefore, our study emphasizes that in Kuwait, health providers and institutions have not developed enough education programs about sun exposure hazards. This development must be started in schools, universities, institutions, and companies, especially those with outdoor workers.

The lack of knowledge about skin cancer in Kuwait can be attributed to several different factors. In Kuwait, skin cancer is not frequently reported, and studies have demonstrated a significantly low incidence of non-melanoma skin malignancies, including basal cell carcinoma and squamous cell carcinoma.^1^^,^^20^ For example, a 13-year review at a major dermatology center found only 146 skin cancer cases out of 7645 skin pathology reports, with the disease incidence not exceeding 1.9% during the period.^1^ Public awareness and healthcare provider focus are typically lower when a disease is less prevalent in the population. The majority of Kuwait's population has skin types that are less susceptible to skin cancer than lighter-skinned populations. Cultural practices, such as wearing traditional clothing that covers much of the body, reduce direct skin exposure to the sun. This further lowers the perceived personal risk of skin cancer and may contribute to less public discussion or education regarding the disease. Several recent publications have emphasized the scarcity of local studies and data on cutaneous cancer in Kuwait.^1^^,^^20^ The shortage of research results in reduced media coverage, fewer educational campaigns, and limited incorporation of public health priorities. Public education efforts on skin cancer and sun protection are not as widespread or emphasized as other health concerns, further contributing to lower knowledge levels.

## Conclusion

Due to Kuwait's high UV index and hot climate, it is imperative that residents adhere to solar safety protocols, which include the use of sunscreen. The adult Kuwaiti participants possessed a satisfactory level of awareness regarding the adverse effects of the sun on their skin, except the knowledge of skin cancer which was low. Furthermore, a substantial proportion of the population implements proactive strategies to reduce their exposure to sunlight. Nevertheless, Kuwaiti adults' utilization of sunscreen was inadequate, regardless of their awareness and education. In Kuwait, future research must improve solar protection practices and mitigate sun-related skin damage.

## Conflicts of interest

None disclosed.
